# Factors Associated with Bone Health in Malaysian Middle-Aged and Elderly Women Assessed via Quantitative Ultrasound

**DOI:** 10.3390/ijerph14070736

**Published:** 2017-07-06

**Authors:** Kok-Yong Chin, Nie Yen Low, Wan Ilma Dewiputri, Soelaiman Ima-Nirwanaa

**Affiliations:** 1 Department of Pharmacology, Universiti Kebangsaan Malaysia Medical Centre, Cheras 56000, Malaysia; imasoel@ppukm.ukm.edu.my; 2 ASASIpintar, PERMATApintar National Gifted Centre, Universiti Kebangsaan Malaysia, Bangi 43600, Malaysia; nicolelow97@gmail.com (N.Y.L.); ilma.dewiputri@ukm.edu.my (W.I.D.)

**Keywords:** calcaneus, menopause, parity, osteopenia, osteoporosis, risk factor

## Abstract

Risk factors for osteoporosis may vary according to different populations. We aimed to investigate the relationship between risk factors of osteoporosis and bone health indices determined via calcaneal quantitative ultrasound (QUS) in a group of Malaysian women aged 50 years or above. A cross-sectional study was performed on 344 Malaysian women recruited from a tertiary medical centre in Kuala Lumpur, Malaysia. They answered a self-administered questionnaire on their social-demographic details, medical history, lifestyle, and physical activity status. Their height was measured using a stadiometer, and their body composition estimated using a bioelectrical impedance device. Their bone health status was determined using a water-based calcaneal QUS device that generated three indices, namely speed of sound (SOS), broadband ultrasound attenuation (BUA), and stiffness index (SI). A T-score was computed from SI values using a reference database from a mainland Chinese population. Women with three or more lifetime pregnancies, who were underweight and not drinking coffee had a significantly lower BUA. Stepwise multiple linear regression showed that SOS was predicted by age alone, BUA and SI by years since menopause, body mass index (BMI), and number of lifetime pregnancies, and T-score by years since menopause and percentage of body fat. As a conclusion, suboptimal bone health in middle-aged and elderly Malaysian women as indicated by QUS is associated with old age, being underweight, having a high body fat percentage, and a high number of lifetime pregnancies. Women having several risk factors should be monitored more closely to protect their bones against accelerated bone loss.

## 1. Introduction

Aging of the female skeletal system accelerates at the commencement of menopause [1]. This event is driven by a halt in the production of oestrogen, which is essential in maintaining bone health in women [2]. The imbalance in bone homeostasis, which favours resorption over formation, leads to deterioration of bone microarchitecture and mass, and ultimately results in a skeleton with reduced strength which is more prone to fragility fractures [3]. This condition is known as post-menopausal osteoporosis. Although menopause is universal among women, post-menopausal osteoporosis is not. Several modifiable and non-modifiable risk factors predispose women to osteoporosis. Being underweight, parity, a sedentary lifestyle, cigarette smoking, alcohol and caffeine intake, as well as low calcium consumption are modifiable risk factors known to affect bone health, whereas old age and ethnicity are examples of non-modifiable ones [1,4,5]. Although these factors are well-established, the interplay between them and bone health could vary from population to population.

Early screening enables women to take preventive actions to minimize bone loss. A dual-energy X-ray absorptiometry device (DEXA) is the most common means of measuring bone mineral density (BMD) [6]. However, in developing countries like Malaysia, DEXA is reserved for the purpose of diagnosis and monitoring treatment of osteoporosis instead of screening [7]. Quantitative ultrasound (QUS) devices offer an alternative solution to mass bone health screening because it is inexpensive, portable, and free from ionising energy [8]. The calcaneus is the site of measurement recommended by the International Society of Clinical Densitometry [9]. Previous studies have established that QUS indices correlate strongly with BMD and are predictive of fractures [10,11]. In Malaysia, a series of studies have been performed to determine the association between calcaneal speed of sound with anthropometric, biochemical, and metabolic indices in men [12,13,14]. Calcaneal speed of sound measures the velocity of ultrasound waves traveling across the calcaneal bone [8]. Since sound waves propagate faster in denser objects, a higher speed of sound value indicates a denser bone [8]. Several studies involving women are available but they were limited in scope and sample size [15,16].

In a previous study, we established that 43.4% of Malaysian women aged 50 years or above who underwent a bone health screening in a tertiary referred hospital were at medium to high risk of osteoporosis as indicated by a QUS device [17]. The aim of the present study was to investigate the relationship between socio-demographic, anthropometric, and lifestyle risk factors for osteoporosis and QUS indices in the same group of women. We hoped this study could highlight the risk factors associated with bone health in Malaysian women at risk for osteoporosis, so that proactive action could be considered to minimize bone loss in potentially high-risk individuals.

## 2. Materials and Methods

A cross-sectional study was performed from 1 December 2014 to 31 November 2015 at a tertiary referral hospital in central Malaysia. Subjects were recruited onsite without prior invitation via a purposive sampling method, which is a form of convenient sampling method with pre-determined inclusion and exclusion criteria. They were female visitors (patients on follow-up and accompanying persons of the patients) of the hospital aged 50 years or above. Subjects fulfilling any of the following criteria were excluded: (1) previously diagnosed with osteoporosis, osteomalacia, or osteogenesis imperfecta; (2) currently receiving treatment for osteoporosis (hormone replacement therapy, bisphosphonates, strontium ranelate, denosumab, or teriparatide); (3) currently receiving medications affecting bone health, such as hormone deprivation therapy, glucocorticoids, or thyroid supplements.; (4) having mobility problems, or metal implants in their lower limbs. Subjects were provided with details of the study and written consent was obtained before enrolling them in the study. The study protocol was reviewed and approved by the Research Ethics Committee of Universiti Kebangsaan Malaysia (project code: FF-2015-412).

The subjects answered a questionnaire on their social demographic details, lifestyle, and physical activity status. Age and sex of the subjects were determined from the records on their identification card. Ethnicity, education level, number of lifetime pregnancies, and age of menarche and menopause were self-declared. The subjects were requested to disclose their cigarette-smoking habits and alcohol, milk, and coffee intake. For beverages, an intake of less than 1 unit per week was defined as non-drinker. Alcohol unit was defined according to the recommendation by National Health Service, UK [18]. One unit of milk was defined as 200 mL whereas coffee was defined as one standard tea cup. Due to the low number of subjects who ceased smoking (*n* = 1) or consuming alcohol (*n* = 1) and coffee (*n* = 1), ex-users and current users were combined to form ‘ever-smokers’ or ‘ever-drinkers’ (Table 1).

Physical activity status of the subjects was determined using a self-administered International Physical Activity Questionnaire (IPAQ) (short form), which is freely available online [19]. Briefly, the subjects were required to note down the time spent and frequency of walking, as well as moderate and vigorous physical activities in a week. These were converted to metabolic equivalent of task (MET) and summed up. Subjects were classified into inactive, minimally active, or HEPA (health-enhancing physical activity) active based on the total MET score or other additional criteria. This questionnaire has been used and validated in the Malay population [20].

Standing height of the subjects without shoes was measured to the nearest 1 cm using a stadiometer (Seca, Hamburg, Germany). Body composition was measured using a SC-330 Body Composition Analyser (Tanita, Arlington Heights, IL, USA) based on the bioelectrical impedance principle. Its short-term in vivo coefficient of variation for the measurement of body fat percentage was around 1%. Body weight was recorded to the nearest 0.1 kg. Body mass index (BMI; kg/m^2^) was calculated as per convention. Bone health was determined using an Achilles EXPII (GE Healthcare UK Ltd., Little Chalfont, UK), a water-based calcaneal QUS device. Subjects placed their right foot in the foot pad of the device in a sitting position. Ultrasound waves were transmitted from water-inflated transducer through the calcaneus and received by another transducer and were analysed. Three measurements with repositioning were taken and the averaged values were used in the analysis. The device generates three ultrasound parameters, i.e., speed of sound (SOS), broadband ultrasound attenuation (BUA), and stiffness index (SI), which is a composite parameter ([0.67 × BUA] + [0.28 × SOS] − 420). By definition, SOS is the time taken for ultrasound waves to travel through the calcaneus, whereas BUA is the slope of attenuation of the ultrasound signals. Denser bones transmit ultrasound waves faster (indicated by a higher SOS value) and attenuate ultrasound signals at higher frequency (indicated by a higher BUA value), thus resulting in a higher SI value. The devise also generates T-score based on SI values with reference to a mainland Chinese population as a local reference is not available. The QUS device was handled by trained technicians. Calibration was performed at the beginning of each screening session. The short-term in vivo coefficient of variation for the device was <2%.

### Statistical Analysis

Normality of the data was determined using the Kolmogorov-Smirnov test. Square root transformation was performed for BUA values, whereas logarithm transformation was performed for BMI values to improve their distribution. Comparison of the mean of QUS indices across the study groups was performed using univariate analysis with adjustment for age and/or BMI because they are potential confounding factors. Pair-wise comparison was performed using Sidak test. Multiple linear regression was performed to select the best predictors of QUS indices. A two-step model was used to identifying the best predictors of QUS indices. The first step was a stepwise regression model to select the best continuous variables. The second step involved forced entry of dummy coded categorical predictors that were not entered in the first step. However, none of the categorical predictors were statistically significant in the second step in this study. Thus, only results of the first step are shown. Statistical analysis was executed using Statistical Software for Social Sciences version 20.0 (IBM, Armonk, NY, USA). Statistical significance was set at *p* < 0.05.

## 3. Results

A total of 459 women volunteered for the study, but 35 were excluded for taking hormone replacement therapy, 26 for osteoporosis treatment, 7 for glucocorticoids, 28 for thyroid supplements, and 19 for not completing the screening process. Data from the remaining 344 women (mean age 61.8 years; standard deviation 7.6 years) were included in the analysis. The ethnic composition of the subjects was 57.3% Malay, 34.6% Chinese, and 8.1% Indian and others (Table 1). The age and height of Chinese women were significantly higher, whereas their body weight, BMI, and body fat percentage were significantly lower compared to Malay women (*p* < 0.05) (Data not shown). However, there were no significant differences in years since menopause and QUS indices among the three ethnic groups (*p* > 0.05) (Table 2).

For the categorical variables, women with more than three lifetime pregnancies had a lower BUA compared to those who were nulliparous or had one to three pregnancies previously (*p* < 0.05). Women classified as underweight (BMI < 18.5 kg/m^2^) had a significantly lower BUA compared to all other BMI categories (*p* < 0.05). In addition, obese women (BMI ≥ 30 kg/m^2^) had a higher BUA compared to women with normal BMI (between 18.5 kg/m^2^ and 24.9 kg/m^2^) (*p* < 0.05). There were no significant differences in other QUS indices across BMI categories (*p* > 0.05). Coffee drinkers had a significantly higher BUA compared to non-drinkers (*p* = 0.014), but this was not shown in other QUS indices. Other factors, such as ethnicity, education level, physical activity status, cigarette-smoking, alcohol, and milk intake did not affect QUS indices significantly (*p* > 0.05). All comparisons were adjusted for age and BMI (Table 2).

Stepwise multiple regression analysis showed that age alone was the significant negative predictor of SOS (β = −0.299, *p* < 0.001) (*n* = 344). Years since menopause (β = −0.306, *p* < 0.001) and number of lifetime pregnancies (β = −0.133, *p* = 0.011) were negative predictors, and BMI (log-transformed) (β = 0.242, *p* < 0.001) was a positive predictor of BUA (*n* = 320). Years since menopause (β = −0.358, *p* < 0.001) and number of lifetime pregnancies (β = −0.112, *p* = 0.033) were negative predictors, and BMI (log-transformed) (β = 0.157, *p* = 0.003) was a positive predictor of SI. Years since menopause (β = −0.356, *p* < 0.001) and percentage of body fat (β = −0.148, *p* = 0.004) were negative predictors of T-score for women in this study (Table 3).

## 4. Discussion

The current study utilized a QUS device that generated three different indices, i.e., SOS, BUA, and SI. T-score was computed by comparing the SI values of the subjects with the reference from a mainland Chinese population. Each QUS index was influenced by a distinct subset of factors associated with bone health. All indices decreased significantly with increasing age. Increased years since menopause, higher number of pregnancy, and decreased BMI were related with decreased BUA and SI. Coffee intake was associated with increased BUA. Apart from years since menopause, elevated percentage of body fat was linked with decreased T-score in Malaysian women. Other factors were not associated with the QUS indices studied. Earlier studies demonstrated that QUS detects variation in bone quality apart from mass, such as strength and trabecular microarchitecture [8,21,22]. Factors influencing each aspect of bone quality may be different, thus explaining the difference in their degree of association with distinct QUS indices.

Age is a major predictor of bone health in women. Women experience two phases of bone loss characterized by an accelerated phase immediately after menopause, and a gradual phase at a later stage of life [23]. The initial rapid bone loss can be attributed to cessation of ovarian oestrogen production at the onset of menopause, whereas the gradual phase is regarded as senile bone loss common to both sexes [24]. The linear age trend of QUS indices in this study reflected the gradual bone loss in elderly women. Since younger women were not recruited, the accelerated bone loss during menopause cannot be depicted due to the lack of a comparison group. The negative relationship between age and bone health indicated by BMD or QUS indices was shown in other epidemiological studies as well [25,26,27].

Years since menopause indicated how long a postmenopausal woman was deprived of oestrogen. Without the protective action of oestrogen, there will be a progressive increase in bone resorption and a decrease in bone formation, leading to deterioration of bone microarchitecture and strength [28]. In line with this, an increase in years since menopause in the women of our study was associated with a reduction in QUS indices. In fact, it was a stronger predictor for BUA, SI, and T-score than chronological age in multiple linear regression analysis. The negative association between years since menopause and bone health was also observed in other studies [27,29,30]. Considering the negative association between chronological age/years since menopause and bone health, postmenopausal elderly women are at an increased risk for osteoporosis. This necessitates them to undergo annual BMD assessment to enable early diagnosis and treatment of osteoporosis. In fact, the Malaysian Clinical Guidelines for Management of Osteoporosis indicates that all women aged 65 years and above should have annual BMD assessments [31].

Another gynaecological index related to bone health is the number of lifetime pregnancies (parity). Evidence on the relationship between parity and bone health is heterogeneous, whereby both positive and negative relationships have been reported [25,27,32]. The latest meta-analysis indicated that an increase in the number of lifetime pregnancies was associated with reduced hip fracture [12% (95% confidence interval: 9–15%) for each live birth] and reduced osteoporotic fracture [25% (95% confidence interval: 16–33%) for five live births] [33]. This disagrees with our observation which showed that increased number of lifetime pregnancies was associated with lower BUA and SI values. Møller et al. demonstrated that pregnancy could cause a reversible decline of BMD, which could be compounded by breastfeeding [34]. After 19 months, the BMD of the mother returns to normal [34]. The Study of Women’s Health Across the Nation (SWAN) demonstrated that despite the positive effects of parity on bone strength, accumulated length of lactation was negatively associated with BMD at the lumbar spine [35]. We speculate that narrow gaps between pregnancies and poor nutrition could explain the observation in this study. However, data on breastfeeding, interval between pregnancies, and post-partum nutrition were not collected in this study. Thus, this speculation awaits further validation.

Ethnic differences in bone health have been reported in multiracial populations. In the United States, African American women were found to have a higher BMD and lower fracture rates compared to their Hispanic and Caucasian counterparts [36,37]. Similarly, African women had a higher BMD compared to the Caucasian women in South Africa [38]. However, differences in QUS indices were not significant among Chinese, Malay, and Indian women in this study. This was supported by a previous study in Malaysia, whereby BMD was found to be similar among middle-aged urban-dwelling Chinese, Malay, and Indian women [39]. A study on Malaysian men also showed that SOS values between Chinese and Malays were similar across age groups [14]. Difference in hip fracture incidence among Chinese, Malay, and Indians in Malaysia had been reported [40]. This disparity could not be explained using BMD and bone quality as reflected by QUS. Non-BMD factors, such as muscle strength and a tendency to fall, could be responsible for ethnic differences in fracture risk [41].

Increased BMI was associated with increased BUA and SI of the subjects in this study. Body mass index is reflective of the body loading onto the bone. The skeleton responds to mechanical loading by increasing its mass [42]. Thus, higher BMD values or QUS indices in subjects with higher BMI was a common finding in previous epidemiological studies [26,27,43,44]. However, BMI is not the most accurate obesity index [45]. In our study, fat mass was determined using a bioelectrical impedance instrument. T-score of our subjects showed a negative relationship with percentage body fat. This implies that increased body fat could oppose the protective effects of mechanical loading on bone exerted by large body size. Production of cytokines by the adipose tissue, coupled with higher oxidative stress levels among the obese individuals might be responsible for the negative effects of fat on bone [46]. Vitamin D, an important nutrient for bone health, is often reported to be low in obese individuals [47]. This could be due to the lack of physical activity and sunlight exposure, or the sequestration of vitamin D by adipose tissue, rendering it unavailable for bone homeostasis [48,49]. These could explain the negative association between fat mass and bone in this study. A similar negative association between fat and bone health has been observed by other researchers [50,51]. However, the dynamic between fat mass and bone health is complicated. Positive relationships between fat mass and bone mineral density and bone strength have also been reported [52,53].

Physical activity, especially weight-bearing activity, have been shown to maintain optimal bone health by exerting mechanical loading onto the bone [54]. However, QUS indices among women with different physical activity statuses did not differ statistically in this study. This could be attributed to the nature of IPAQ (short form) which does not differentiate between weight-bearing and non-weight-bearing activities. It is also possible that lifetime physical activities, especially during acquisition of peak bone mass, are more important in determining bone health in later life compared to recent physical activities [55]. Nicotine in cigarettes is harmful to the bone, and cigarette smoking was associated with low BMD in several epidemiological studies [56,57,58,59]. However, the effect size of smoking on QUS indices could be small, thus the difference between smokers and non-smokers was not apparent in the current study. We did not explore the dose-dependent effects of cigarette-smoking on bone due to the lack of information on the exposure level among our subjects. Although coffee consumption has been suggested as a risk factor of osteoporosis, several large epidemiological studies reported that the association was marginal at best [60]. In this study, we found that coffee drinkers had a higher BUA compared to non-drinkers. This is supported by recent studies showing that moderate coffee intake (<3 cups per day) was associated with increased BMD and reduced risk for osteoporosis in Asians [61,62]. Although caffeine might be detrimental to bone, other polyphenols in coffee possessing oestrogenic, antioxidant, and anti-inflammatory properties that are possibly beneficial to bone could contribute to this positive association [63,64,65]. In addition, no significant differences in all QUS indices were detected between milk drinkers and non-takers. The median intake of milk was one glass a day, which might be insufficient to exert bone beneficial effects. Only a small number of the subjects were consuming alcohol, thus we were not able to detect any differences if present.

Several limitations of this study should be considered carefully. Firstly, the causal relationship between bone health and risk factors of osteoporosis cannot be established in this cross-sectional design. Subjects were recruited using a non-randomized sampling method in a hospital setting, thus, generalization of the results might be difficult. The questionnaire was self-administered, therefore, recall bias was possible and it might affect the accuracy of the results. Vitamin D insufficiency, which could have a negative impact on bone health, is reported to be prevalent in Malaysians [49,66], however, it was not examined in this study. Nevertheless, this study could serve as a pilot for larger and more comprehensive longitudinal studies in the future to establish the causal relationship between the observed risk factors and bone loss.

## 5. Conclusions

Bone health of Malaysian women as depicted by QUS indices is negatively associated with increased chronological age, years since menopause, number of lifetime pregnancies, percentage of body fat, and suboptimal BMI. Therefore, postmenopausal multiparous elderly Malaysian women who are underweight should undergo regular BMD assessments to prevent osteoporosis and its associated fractures via early diagnosis and treatment.

## Figures and Tables

**Table 1:** Characteristics of the subjects.

Variable of Interest		*n*	Mean	Standard Deviation	Notes
Age (years)		344	61.8	7.6	
Age of menarche (years)		335	13.3	1.7	9 could not recall the age of menarche
Age of menopause (years)		327	49.9	5.8	17 had not reached menopause
Years since menopause (years)		327	11.9	9.4	
Weight (kg)		344	60.5	11.3	
Height (cm)		344	153.7	5.7	
BMI (kg/m^2^)		344	25.7	4.7	
Body fat percentage (%)		344	36.2	7.0	
Speed of sound (m/s)		344	1536.0	28.6	
Broadband attenuation of sound (dB/MHz)		344	112.4	11.7	
Stiffness index		344	84.8	14.5	
T-score		344	−0.7	1.4	
Total MET		344	2922.0	2046.8	
Number of children (*n*)		344	2.9	1.8	
		*n*	%		
Ethnicity	Chinese	119	34.6		
	Malay	197	57.3		
	Indian	28	8.1		
Menopause status	Natural menopause	274	79.7		
	Menopause due to surgery	41	11.9		
	Menopause due to drugs	12	3.5		
	Perimenopausal	17	5		
Education level	No formal education	17	4.9		
	Primary school	60	17.4		
	Secondary school	168	48.8		
	Certificate	31	9		
	Diploma	37	10.8		
	Degree	23	6.7		
	Postgraduate	8	2.3		
Cigarette smoking status	Non-smoker	335	97.4		
	Current smoker	8	2.3		
	Ex-smoker	1	0.3		
Alcohol drinking	Non-drinker	336	97.7		
	Current drinker	7	2.0		
	Ex-drinker	1	0.3		
Milk drinking	Non-drinker	173	50.3		
	Drinker	171	49.7		
Coffee drinking	Non-drinker	146	42.4		
	Current drinker	197	57.3		
	Ex-drinker	1	0.3		
Physical activity status	Inactive	15	4.4		
	Minimally active	193	56.1		
	HEPA active	136	39.5		

BMI = body mass index; MET = metabolic equivalent of task; HEPA = health-enhancing physical activity.

**Table 2:** Quantitative ultrasound (QUS) indices of the categorical variables. SOS = speed of sound; BUA = broadband ultrasound attenuation; SI = stiffness index.

Variable		SOS (m/s) *			BUA (dB/MHz) *^,#^			SI *			T-Score *		
		Mean	SE	*p*-Value	Mean	SE	*p*-Value	Mean	SE	*p*-Value	Mean	SE	*p*-Value
Ethnicity	Malay	1531.228	2.595	0.083	111.09	1.037	0.171	82.735	1.294	0.083	−0.88	0.128	0.104
	Chinese	1538.602	1.998		113.435	0.798		86.354	0.997		−0.524	0.099	
	Indian/Punjabi	1538.204	5.149		110.744	2.058		83.106	2.568		−0.655	0.254	
BMI	Underweight (<18.5 kg/m^2^)	1528.125	11.24	0.747	99.009	4.491	<0.001	73.879	5.617	0.053	−1.789	0.555	0.042
	Normal (18.5–24.9 kg/m^2^)	1534.8	2.193		111.005	0.876	a	83.512	1.096		−0.782	0.108	
	Overweight (25–29.9 kg/m^2^)	1537.188	2.431		113.168	0.971	a	85.961	1.215		−0.573	0.12	
	Obese (≥30 kg/m^2^)	1537.637	3.7		116.07	1.478	a,b	87.2	1.849		−0.38	0.183	
Menopause status	Natural menopause	1535.652	1.654	0.544	112.213	0.659	0.465	84.782	0.823	0.315	−0.675	0.082	0.529
	Menopause due to surgery	1536.483	4.285		112.895	1.708		84.529	2.133		−0.622	0.211	
	Menopause due to medications	1530.755	7.923		110.026	3.158		80.255	3.944		−0.968	0.391	
	Perimenopausal	1544.531	6.675		115.986	2.66		89.71	3.322		−0.255	0.329	
Education level	No formal education	1535.082	6.948	0.987	110.603	2.759	0.611	81.202	3.455	0.746	−0.818	0.342	0.878
	Primary	1533.733	3.651		110.948	1.45		83.646	1.815		−0.787	0.18	
	Secondary	1536.355	2.124		112.31	0.843		84.838	1.056		−0.661	0.105	
	Certificate	1537.337	4.964		114.226	1.971		86.609	2.468		−0.51	0.244	
	Diploma	1535.834	4.561		112.415	1.811		84.944	2.268		−0.673	0.225	
	Degree or above	1538.034	5.004		114.893	1.987		87.241	2.488		−0.434	0.246	
Number of lifetime pregnancies	nulliparous	1540.789	4.228	0.458	114.611	1.668	0.01	87.73	2.099	0.097	−0.388	0.208	0.114
	1–3	1535.809	2.016		113.44	0.795		85.449	1.001		−0.597	0.099	
	>3	1534.638	2.553		109.975	1.007	a,b	82.833	1.268		−0.851	0.125	
Physical activity status	Inactive	1529.842	7.108	0.373	109.179	2.837	0.468	81.164	3.549	0.512	−1.058	0.351	0.446
	Minimally active	1537.672	1.965		112.552	0.784		85.298	0.981		−0.608	0.097	
	HEPA active	1534.354	2.347		112.55	0.937		84.59	1.172		−0.684	0.116	
Smoking status	Non-smoker	1536.054	1.493	0.884	112.45	0.595	0.583	84.875	0.745	0.760	−0.653	0.074	0.710
	Ever-smoker	1534.699	9.167		110.699	3.655		83.459	4.572		−0.824	0.452	
Alcohol drinking	Non-drinker	1536.034	1.491	0.946	112.338	0.594	0.454	84.793	0.744	0.696	−0.662	0.074	0.729
	Ever-drinker	1535.367	9.712		115.19	3.87		86.712	4.843		−0.494	0.479	
Milk drinking	Non-drinker	1534.362	2.082	0.262	112.227	0.832	0.748	84.167	1.039	0.362	−0.711	0.103	0.464
	Drinker	1537.695	2.094		112.584	0.837		85.517	1.045		−0.604	0.103	
Coffee drinking	Non-drinker	1535.348	2.254	0.695	110.938	0.893	0.014	83.555	1.121	0.132	−0.776	0.111	0.160
	Ever-drinker	1536.519	1.947		113.499	0.771	a	85.795	0.968		−0.57	0.096	

Legend: * all comparisons were adjusted with age and BMI; ^#^ square-root transformed values were used in the analysis but actual mean values are displayed in the table; a = significant difference (*p* < 0.05) compared to the first group in the category; b = compared to the second group in the category.

**Table 3:** Stepwise multiple linear regression between QUS indices and variables of interest.

Dependent Variable	Independent Variable					R^2^ Model
		Unstandardized Coefficients		Standardized Coefficients	*p*-Value	
		B	Standard Error	Beta		
Speed of sound (m/s) (*n* = 344)	Constant for model	1606.007	12.288		<0.001	0.090
	Age (years)	−1.131	0.197	−0.299	<0.001	
Broadband ultrasound attenuation (square-root transformed) (dB/MHz) (*n* = 320)	Constant for model	8.502	0.518		<0.001	0.176
	Years since menopause (years)	−0.018	0.003	−0.306	<0.001	
	BMI (log-transformed) (kg/m^2^)	1.711	0.370	0.242	<0.001	
	Number of lifetime pregnancies (*n*)	−0.040	0.016	−0.133	0.011	
Stiffness index (*n* = 319)	Constant for model	52.746	13.581		<0.001	0.174
	Years since menopause (years)	−0.562	0.081	−0.358	<0.001	
	BMI (log-transformed) (kg/m^2^)	28.992	9.692	0.157	0.003	
	Number of of lifetime pregnancy (*n*)	−0.888	0.414	−0.112	0.033	
T-score (*n* = 318)	Constant for model	1.858	0.680		0.007	0.158
	Years since menopause (years)	−0.054	0.008	−0.356	<0.001	
	Percentage of body fat (%)	−0.030	0.011	−0.148	0.004	

## References

[1] Curtis E., Litwic A., Cooper C., Dennison E. (2015). Determinants of muscle and bone aging. J. Cell. Physiol..

[2] Khosla S., Oursler M.J., Monroe D.G. (2012). Estrogen and the skeleton. Trends Endocrinol. Metab..

[3] Khosla S. (2010). Update on estrogens and the skeleton. J. Clin. Endocrinol. Metab..

[4] Schnatz P.F., Marakovits K.A., O’Sullivan D.M. (2010). Assessment of postmenopausal women and significant risk factors for osteoporosis. Obstet. Gynecol. Surv..

[5] ToRo K.O.R. (2013). A study of risk factors and t- score variability in Romanian women with postmenopausal osteoporosis. Iran. J. Public Health.

[6] World Health Organization (1994). Assessment of Fracture Risk and Its Application to Screening for Postmenopausal Osteoporosis: Report of a World Health Organization Study Group.

[7] Mithal A., Ebeling P. (2013). The Asia-Pacific Regional Audit: Epidemiology, Costs & Burden of Osteoporosis in 2013.

[8] Chin K.Y., Ima-Nirwana S. (2013). Calcaneal quantitative ultrasound as a determinant of bone health status: What properties of bone does it reflect?. Int. J. Med. Sci..

[9] Krieg M.A., Barkmann R., Gonnelli S., Stewart A., Bauer D.C., Del Rio Barquero L., Kaufman J.J., Lorenc R., Miller P.D., Olszynski W.P. (2008). Quantitative ultrasound in the management of osteoporosis: The 2007 iscd official positions. J. Clin. Densitom..

[10] Moayyeri A., Adams J., Adler R., Krieg M.A., Hans D., Compston J., Lewiecki E. (2012). Quantitative ultrasound of the heel and fracture risk assessment: An updated meta-analysis. Osteoporos. Int..

[11] Chan M.Y., Nguyen N.D., Center J.R., Eisman J.A., Nguyen T.V. (2013). Quantitative ultrasound and fracture risk prediction in non-osteoporotic men and women as defined by who criteria. Osteoporos. Int..

[12] Chin K.-Y., Soelaiman I.-N., Mohamed I.N., Ibrahim S., Ngah W.Z.W. (2012). The effects of age, physical activity level, and body anthropometry on calcaneal speed of sound value in men. Arch. Osteoporos..

[13] Chin K.-Y., Soelaiman I.-N., Mohamed I.N., Wan Ngah W.Z. (2012). Serum testosterone, sex hormone-binding globulin and total calcium levels predict the calcaneal speed of sound in men. Clinics.

[14] Chin K.Y., Soelaiman I.N., Mohamed I.N., Mohamed N., Shuid A.N., Muhammad N., Wan Ngah W.Z. (2013). Discrepancy between the quantitative ultrasound value of Malaysian men and the manufacturer’s reference and the impact on classification of bone health status. J. Clin. Densitom..

[15] Chan P.J., Nurul Z.Z., Chuah J.S., Nabil M.M.A., Isa N.M., Sabarul A.M., Nazrun A.S. (2014). Association between risk factors of osteoporosis and bone mineral density in women of different ethnic groups in a Malaysian hospital. Int. J. Osteoporos. Metab. Disord..

[16] Hasnah H., Amin I., Suzana S. (2012). Bone health status and lipid profile among post-menopausal malay women in Cheras, Kuala Lumpur. Malays. J. Nutr..

[17] Chin K.-Y., Kamaruddin A.A.A., Low N.Y., Ima-Nirwana S. (2016). Effects of age, sex, and ethnicity on bone health status of the elderly in Kuala Lumpur, Malaysia. Clin. Interv. Aging.

[18] National Health Service Alcohol Units. http://www.nhs.uk/Livewell/alcohol/Pages/alcohol-units.aspx.

[19] The International Physical Activity Questionnaire Group Downloadable IPAQ Questionnaires. https://sites.google.com/site/theipaq/questionnaire_links.

[20] Chu A.H., Moy F.M. (2015). Reliability and validity of the Malay international physical activity questionnaire (IPAQ-M) among a Malay population in Malaysia. Asia Pac. J. Public Health.

[21] Haiat G., Padilla F., Peyrin F., Laugier P. (2007). Variation of ultrasonic parameters with microstructure and material properties of trabecular bone: A 3d model simulation. J. Bone Miner. Res..

[22] Qin Y.-X., Lin W., Mittra E., Xia Y., Cheng J., Judex S., Rubin C., Müller R. (2013). Prediction of trabecular bone qualitative properties using scanning quantitative ultrasound. Acta Astronaut..

[23] Yuen K.W., Kwok T.C., Qin L., Leung J.C., Chan D.C., Kwok A.W., Woo J., Leung P.C. (2010). Characteristics of age-related changes in bone compared between male and female reference Chinese populations in Hong Kong: A PQCT study. J. Bone Miner. Metab..

[24] Cauley J.A. (2015). Estrogen and bone health in men and women. Steroids.

[25] Adami S., Giannini S., Giorgino R., Isaia G., Maggi S., Sinigaglia L., Filipponi P., Crepaldi G., Di Munno O. (2003). The effect of age, weight, and lifestyle factors on calcaneal quantitative ultrasound: The ESOPO study. Osteoporos. Int..

[26] Ding Z., Chen Y., Xu Y., Zhou X., Xu Y., Ma Z., Sun Y. (2016). Impact of age, gender, and body composition on bone quality in an adult population from the middle areas of china. J. Clin. Densitom..

[27] El Maghraoui A., Guerboub A.A., Mounach A., Ghozlani I., Nouijai A., Ghazi M., Achemlal L., Bezza A., Tazi M.A. (2007). Body mass index and gynecological factors as determinants of bone mass in healthy Moroccan women. Maturitas.

[28] Brennan O., Kuliwaba J.S., Lee T.C., Parkinson I.H., Fazzalari N.L., McNamara L.M., O’Brien F.J. (2012). Temporal changes in bone composition, architecture, and strength following estrogen deficiency in osteoporosis. Calcif. Tissue Int..

[29] Assantachai P., Sriussadaporn S., Thamlikitkul V., Sitthichai K. (2006). Body composition: Gender-specific risk factor of reduced quantitative ultrasound measures in older people. Osteoporos. Int..

[30] Gemalmaz A., Discigil G., Sensoy N., Basak O. (2007). Identifying osteoporosis in a primary care setting with quantitative ultrasound: Relationship to anthropometric and lifestyle factors. J. Bone Miner. Metab..

[31] Society M.O. (2015). Clinical Guidance on Management of Osteoporosis 2012.

[32] Mahboub S.M., Al-Muammar M.N., Elareefy A.A. (2014). Evaluation of the prevalence and correlated factors for decreased bone mass density among pre- and post-menopausal educated working women in Saudi Arabia. J. Health Popul. Nutr..

[33] Wang Q., Huang Q., Zeng Y., Liang J.J., Liu S.Y., Gu X., Liu J.A. (2016). Parity and osteoporotic fracture risk in postmenopausal women: A dose-response meta-analysis of prospective studies. Osteoporos. Int..

[34] Moller U.K., Vieth Streym S., Mosekilde L., Rejnmark L. (2012). Changes in bone mineral density and body composition during pregnancy and postpartum. A controlled cohort study. Osteoporos. Int..

[35] Mori T., Ishii S., Greendale G.A., Cauley J.A., Ruppert K., Crandall C.J., Karlamangla A.S. (2015). Parity, lactation, bone strength, and 16-year fracture risk in adult women: Findings from the study of women’s health across the nation (swan). Bone.

[36] Looker A.C., Melton L.J., Borrud L.G., Shepherd J.A. (2012). Lumbar spine bone mineral density in us adults: Demographic patterns and relationship with femur neck skeletal status. Osteoporos. Int..

[37] Barrett-Connor E., Siris E.S., Wehren L.E., Miller P.D., Abbott T.A., Berger M.L., Santora A.C., Sherwood L.M. (2005). Osteoporosis and fracture risk in women of different ethnic groups. J. Bone Miner. Res..

[38] Conradie M., Conradie M.M., Kidd M., Hough S. (2014). Bone density in black and white South African women: Contribution of ethnicity, body weight and lifestyle. Arch. Osteoporos..

[39] Lim P., Ong F., Adeeb N., Seri S., Noor-Aini M., Shamsuddin K., Hapizah N., Mohamed A., Mokhtar A., Wan H. (2005). Bone health in urban midlife malaysian women: Risk factors and prevention. Osteoporos. Int..

[40] Lee J.-K., Khir A.S.M. (2007). The incidence of hip fracture in Malaysians above 50 years of age: Variation in different ethnic groups. APLAR J. Rheumatol..

[41] Cederholm T., Cruz-Jentoft A.J., Maggi S. (2013). Sarcopenia and fragility fractures. Eur. J. Phys. Rehabil. Med..

[42] Reid I.R. (2002). Relationships among body mass, its components, and bone. Bone.

[43] Constant D., Rosenberg L., Zhang Y., Cooper D., Kalla A.A., Micklesfield L., Hoffman M. (2009). Quantitative ultrasound in relation to risk factors for low bone mineral density in south African pre-menopausal women. Arch. Osteoporos..

[44] Gonnelli S., Caffarelli C., Tanzilli L., Merlotti D., Gennari L., Rossi S., Lucani B., Campagna M.S., Franci B., Nuti R. (2011). The association of body composition and sex hormones with quantitative ultrasound parameters at the calcaneus and phalanxes in elderly women. Calcif. Tissue Int..

[45] Elbassuoni E. (2013). Better association of waist circumference with insulin resistance and some cardiovascular risk factors than body mass index. Endocr. Regul..

[46] Cao J.J. (2011). Effects of obesity on bone metabolism. J. Orthop. Surg. Res..

[47] Trevisan C., Veronese N., Berton L., Carraro S., Bolzetta F., De Rui M., Miotto F., Inelmen E.M., Coin A., Perissinotto E. (2017). Factors influencing serum-hydroxivitamin d levels and other bone metabolism parameters in healthy older women. J. Nutr. Health Aging.

[48] Pesarini J.R., Oliveira R.J., Pessatto L.R., Antoniolli-Silva A., Felicidade I., Nardi N.B., Camassola M., Mantovani M.S., Ribeiro L.R. (2017). Vitamin D: Correlation with biochemical and body composition changes in a southern Brazilian population and induction of cytotoxicity in mesenchymal stem cells derived from human adipose tissue. Biomed. Pharmacother..

[49] Chin K.Y., Ima-Nirwana S., Ibrahim S., Mohamed I.N., Wan Ngah W.Z. (2014). Vitamin D status in malaysian men and its associated factors. Nutrients.

[50] Zhao L.-J., Liu Y.-J., Liu P.-Y., Hamilton J., Recker R.R., Deng H.-W. (2007). Relationship of obesity with osteoporosis. J. Clin. Endocrinol. Metab..

[51] Hsu Y.H., Venners S.A., Terwedow H.A., Feng Y., Niu T., Li Z., Laird N., Brain J.D., Cummings S.R., Bouxsein M.L. (2006). Relation of body composition, fat mass, and serum lipids to osteoporotic fractures and bone mineral density in Chinese men and women. Am. J. Clin. Nutr..

[52] Han G., Chen Y.M., Huang H., Chen Z., Jing L., Xiao S.M. (2017). Fat mass is positively associated with estimated hip bone strength among Chinese men aged 50 years and above with low levels of lean mass. Int. J. Environ. Res. Public Health.

[53] Wang J., Yan D., Hou X., Chen P., Sun Q., Bao Y., Hu C., Zhang Z., Jia W. (2017). Association of adiposity indices with bone density and bone turnover in the Chinese population. Osteoporos. Int..

[54] Shanb A.A., Youssef E.F. (2014). The impact of adding weight-bearing exercise versus nonweight bearing programs to the medical treatment of elderly patients with osteoporosis. J. Fam. Community Med..

[55] Bielemann R.M., Martinez-Mesa J., Gigante D.P. (2013). Physical activity during life course and bone mass: A systematic review of methods and findings from cohort studies with young adults. BMC Musculoskelet. Disord..

[56] Hapidin H., Othman F., Soelaiman I.N., Shuid A.N., Mohamed N. (2011). Effects of nicotine administration and nicotine cessation on bone histomorphometry and bone biomarkers in sprague-dawley male rats. Calcif. Tissue Int..

[57] Baheiraei A., Pocock N.A., Eisman J.A., Nguyen N.D., Nguyen T.V. (2005). Bone mineral density, body mass index and cigarette smoking among Iranian women: Implications for prevention. BMC Musculoskelet. Disord..

[58] Huitron-Bravo G., Denova-Gutierrez E., Talavera J.O., Moran-Villota C., Tamayo J., Omana-Covarrubias A., Salmeron J. (2016). Levels of serum estradiol and lifestyle factors related with bone mineral density in premenopausal Mexican women: A cross-sectional analysis. BMC Musculoskelet. Disord..

[59] Ugurlu U., Nayki U., Nayki C., Ulug P., Kulhan M., Yildirim Y. (2016). Assessment of smoking for low bone mineral density in postmenopausal Turkish women. Wien. Klin. Wochenschr..

[60] Hallstrom H., Byberg L., Glynn A., Lemming E.W., Wolk A., Michaelsson K. (2013). Long-term coffee consumption in relation to fracture risk and bone mineral density in women. Am. J. Epidemiol..

[61] Choi E., Choi K.H., Park S.M., Shin D., Joh H.K., Cho E. (2016). The benefit of bone health by drinking coffee among Korean postmenopausal women: A cross-sectional analysis of the fourth & fifth Korea national health and nutrition examination surveys. PLoS ONE.

[62] Hirata H., Kitamura K., Saito T., Kobayashi R., Iwasaki M., Yoshihara A., Watanabe Y., Oshiki R., Nishiwaki T., Nakamura K. (2016). Association between dietary intake and bone mineral density in Japanese postmenopausal women: The Yokogoshi cohort study. Tohoku J. Exp. Med..

[63] Kitts D.D. (1987). Studies on the estrogenic activity of a coffee extract. J. Toxicol. Environ. Health.

[64] Halvorsen B.L., Carlsen M.H., Phillips K.M., Bohn S.K., Holte K., Jacobs D.R., Blomhoff R. (2006). Content of redox-active compounds (ie, antioxidants) in foods consumed in the united states. Am. J. Clin. Nutr..

[65] Kim J.Y., Kim D.H., Jeong H.G. (2006). Inhibitory effect of the coffee diterpene kahweol on carrageenan-induced inflammation in rats. Biofactors.

[66] Moy F.M., Hoe V.C., Hairi N.N., Vethakkan S.R., Bulgiba A. (2016). Vitamin D deficiency and depression among women from an urban community in a tropical country. Public Health Nutr..

